# Overweight, Obesity, and Lifestyle Behaviors in Immigrants and Native Children in Madrid—ASOMAD Study

**DOI:** 10.3390/nu17122041

**Published:** 2025-06-19

**Authors:** Asmaa Nehari, Alicia Portals-Riomao, Carlos Quesada-González, Augusto G. Zapico, Eva Gesteiro, Marcela González-Gross

**Affiliations:** 1 ImFINE Research Group, Department of Health and Human Performance, Universidad Politécnica de Madrid, 28040 Madrid, Spain; alicia.portals@upm.es (A.P.-R.); carlos.quesada@upm.es (C.Q.-G.); a.gzapico@upm.es (A.G.Z.); marcela.gonzalez.gross@upm.es (M.G.-G.); 2 Physical Exercise and Health Research Network, EXERNET, 18016 Zaragoza, Spain; 3 Department of Language, Arts and Physical Education, Universidad Complutense de Madrid, 28040 Madrid, Spain; 4 Department of Mathematics Applied to Information and Communication Technologies, Universidad Politécnica de Madrid, 28031 Madrid, Spain; 5 Biomedical Research Center of Pathophysiology of Obesity and Nutrition—CIBERobn, Carlos III Health Institute, 28029 Madrid, Spain

**Keywords:** immigrants, childhood obesity, overweight, physical activity and lifestyle

## Abstract

**Background/Objectives**: Overweight and obesity (OW/OB) in immigrant children is a complex multifactorial issue. This work aims to present the OW/OB profile and lifestyle habits of immigrant children and compare them with their native counterparts. **Methods**: A cross-sectional study (ASOMAD) was conducted in a representative sample of children aged 8–12 years in Madrid from 2020 to 2023. Parental origin, physical activity (PA), screen time, adherence to Mediterranean Diet, sleep, and body composition were assessed by validated methods. OW/OB was determined according to IOTF guidelines. *T*-tests, chi-square tests, and two-way ANOVA tests were applied based on the variables’ characteristics. **Results**: A total of 587 children (54% boys, aged 9.54 ± 1.19 y), 33.2% immigrants, were studied. OW/OB rate was higher in immigrants (39.7% boys and 37.4% girls) than in natives (18.0% boys and 17.7% girls) (*p* < 0.05). Immigrant boys were 30.64 ± 8.39 min/day less active than natives (*p* < 0.001). Both male and female immigrant children devoted considerably more weekday hours to screen time than natives (2.76 ± 2.75; 2.02 ± 2.47 vs. 2.09 ± 2.29; 1.32 ± 1.38; *p* < 0.05, respectively). Immigrant children consumed significantly less fish and olive oil and more pasta or rice almost every day (5 or more/week), ate at fast-food restaurants, consumed fewer dairy products and baked goods or pastries for breakfast (*p* < 0.05), and exhibited worse diet quality compared to Spanish ones. **Conclusions**: Immigrant children exhibited a higher prevalence of OW/OB, higher screen time, lower PA time, and ate less fish and olive oil and more pasta than natives. Additional research is required to explore the causes of these issues and enhance the lifestyle within this group.

## 1. Introduction

Overweight and obesity (OW/OB) are among the most prevalent chronic conditions affecting children today, representing a silent rising epidemic that poses a significant public health challenge in the 21st century [1,2,3]. According to the World Health Organization in the European Region (WHO/Europe), one in three primary school children is affected by childhood obesity [4]. A similar situation has been described among children and adolescents in the United States and other countries [5,6]. In Spain, during the two decades leading up to 2010, the prevalence of childhood obesity has remained markedly elevated, with a notable rise observed, particularly among young children between 7 and 13 years [7]. The ALADINO study reported in 2019 that in Spain, OW/OB among children was 23.3% and 17.3%, respectively. The prevalence of obesity was higher in males, whereas overweight was higher in females. In the last wave of the ALADINO study, performed in 2023, a decrease in excess weight in children in Spain, with a prevalence of 20.2% for OW and 15.9% for OB, was observed [8,9]. The increase in the incidence of excess body weight in children has led to a rise in chronic diseases already in childhood (diabetes type 2, non-alcoholic fatty liver disease (NAFLD), and hypertension) [10], as well in adulthood, such as type 2 diabetes mellitus, mental health disorders, cardiovascular diseases, and certain types of cancer [11,12].

In the pediatric population, the etiology of obesity is affected by a multitude of factors, like a sedentary lifestyle, an unhealthy diet, insufficient physical activity (PA), and environmental factors [3]. The immigrant background can serve as an additional risk factor indirectly because of genetic and cultural aspects [13]. Numerous studies have verified the increased risk of developing OW/OB among migrant groups due to acculturation and changes in lifestyle, as immigrants often leave their traditional eating habits in favor of adopting new dietary practices [14]. European Union (EU) data on international migration flows revealed a rise in immigrant numbers in 2022, with 5.1 million people entering the EU from non-EU nations. By 1 January 2023, this number reached 27.3 million non-EU citizens living in EU countries. Although Spain experienced a low immigration rate throughout the 1980s, the situation has changed dramatically over time. In 2023, Spain experienced a net increase in external migration, totaling 642,296 people, with the region of Madrid being the leading destination, attracting 150,469 net migrants [15]. In fact, it was one of four member states that collectively represented 70.6% of the total number of EU immigrants (together with Germany, France, and Italy). At the national level, in the region of Madrid, the gross immigration rate in 2023 was 10.32 per thousand inhabitants [16,17,18].

The scientific literature presents different studies comparing the prevalence of OW/OB between native and immigrant children. At the commencement of the second decade of the 2020s, European scientific literature revealed that the rate of OW/OB in migrant children consistently surpassed that of native children, where many of them classify children based on their parents’ country of origin [19,20]. However, it often lacks clear insights into effectively comprehending the intricate and multifaceted elements that contribute to poorer health in immigrant children [21], and more specifically, after the COVID-19 pandemic. Europe is facing new challenges hosting people from other countries, and accurate data is needed for public health measures. Therefore, this work aims to (a) broaden our knowledge of school-aged immigrant children living in the city of Madrid through the description of the main determinants of OW/OB, (b) compare the lifestyle behaviors, adherence to the Mediterranean Diet (AMD), eating habits, and obesity profiles of these immigrant children versus their Spanish counterparts, and (c) examine the specific influence of school type, and lifestyle determinants and socioeconomic status (SES), and explore their associations with weight status. Comprehending these distinctions is crucial for formulating targeted interventions and strategic policies designed to address the unique requirements of this demographic group thoroughly.

## 2. Materials and Methods

### 2.1. Study Design

The ASOMAD (Physical Activity, Sedentary lifestyle and Obesity in boys and girls in the city of Madrid) study is a descriptive and analytical cross-sectional study whose objective is to obtain a picture of the current situation regarding PA and sedentary lifestyle after the COVID-19 pandemic in a sample of school children aged 8 to 12 in the Spanish capital, Madrid. 

### 2.2. Sampling and Ethics

To achieve the research objectives, multistage random sampling was executed to accomplish the project’s aims. Administratively, Madrid comprises 21 districts, but for the ASOMAD study, these were aggregated into 10 zones by grouping them according to geographic closeness and zones with equivalent economic profiles as per data from the Madrid Community and City Council [22]. According to these data, in Madrid, primary school students are distributed by school type: about 40 enrolled in public schools, another 40% are in semi-private schools, and the remaining 20% go to private schools. An Excel file was created to catalog all primary schools within the 10 clusters, facilitating the random selection process where one school was chosen, with two additional schools as alternatives. Additional levels of selection included the distribution of grades (3rd, 4th, 5th, and 6th) and school ownership (public, semi-private, and private).

The sampling strategy was based on classrooms composed of 25 students, which aligns with the Spanish legal limit for this educational level [23]. Aiming for at least a 70% response rate, the study successfully reached 360 subjects in each sequence. Final sampling adjustments were required, including increasing private and semi-private school participation and utilizing schools from designated districts. In instances of insufficient enrollment in targeted courses, the course selection was altered to maintain sample balance. Data collection was conducted over the academic years 2020/2021, 2021/2022, and 2022/2023 [24].

The study was conducted following the World Medical Association’s Declaration of Helsinki for Biomedical Research (64th General Assembly, Fortaleza, Brazil, October 2013) concerning research involving human subjects [25], as well as the 1997 Oviedo Convention on Human Rights and Biomedicine as set by the Council of Europe [26]. It has been approved by the Ethics Committee of the Universidad Politécnica de Madrid (UPM) for experimental research (number 20200727-1). Children were permitted to participate in the study following the voluntary signing of informed consent by parents or guardians. All children attending selected schools were eligible for inclusion. No child presented severe intellectual disability that would have prevented him/her from responding to the lifestyle questionnaires.

### 2.3. Study Variables

- PA: PA data were obtained using the PAU-7S questionnaire (Physical Activity Unit—7-Item Screener), which is a valid and reliable instrument for measuring the level of PA throughout the week [27]. WHO guidelines were taken into consideration for analysis, where it is advised that children and adolescents should participate in a minimum of 60 min of moderate to vigorous PA daily on average [28].
- Diet assessment: The KIDMED questionnaire (Mediterranean Diet Quality Index) was used. It evaluated the AMD and the consumption frequency of various food items (fruits, vegetables, dairy products, etc.) [29]. It consists of 16 questions; the index classifies diet quality into three levels: scores ≥ 8 indicate optimal AMD, 4–7 suggest improvement is needed, and ≤3 represents very low diet quality [30].
- Sleep duration: Information on sleep duration for both weekdays (WD) and weekends (WK) was gathered through a validated tool, the SHSA (Sleep Habits Survey for Adolescents) [31].
- Sedentary behaviors: To assess the time spent on sedentary behaviors common among children, like watching television, computer use, video games, etc., the SBQ (Sedentary Behaviors Questionnaire) was used [32]. To gain further insight into screen use behaviors in various time contexts, a differentiation between WD and WK screen time patterns was utilized.
- Country of origin: To ascertain the country of origin of the participants, a sociodemographic questionnaire was sent to the parents of the children [33]. This included a question on the country of birth of the parents and their child. Children were considered to be of Spanish origin if both parents were born in Spain, while if at least one of the parents was born in another country, they were considered immigrants. If one parent was born in Spain and the information was missing for the other parent, the child was excluded from the analysis.
- Body composition: It was carried out using the bioimpedance analysis (BIA) (DC-240, MA, TANITA, Tokyo, Japan). It provides information on body water percentage (BW%) and fat mass percentage (FM%). This model incorporated a scale for measuring body weight (kg) as well.
- Height: Measurement of height (cm) by using a Stadiometer model (Seca 217, Hamburg, Germany). Following the guidelines set by the International Society for the Advancement of Kinanthropometry (ISAK), individuals stood without shoes, ensuring their heels, buttocks, and head touched the wall or measurement surface. The body should be straight, with arms resting by the sides. For proper head alignment, the Frankfort plane (an imaginary line from the bottom edge of the eye socket to the top edge of the ear canal) should be parallel to the ground.
- Waist circumference (WC): The WC was measured in the narrowest zone between the lower costal margin (10th rib) and the top of the iliac crest [34] in triplicate using an anthropometric tape model (Seca 201, Hamburg, Germany).
- BMI-z: The BMI, adjusted for age and sex, was calculated using the International Obesity Task Force (IOTF) guidelines. The IOTF BMI-z score is a standardized measure that accounts for age and sex to assess weight status in children and adolescents, utilizing a specific equation to compare BMI relative to population norms [35].
- Waist-to-height ratio (WHtR): It was calculated as WC (cm) divided by height (cm). It serves as a direct indicator of central body fat and is utilized to evaluate health dangers, including cardiovascular and metabolic conditions. The typical threshold for central obesity is having a WHtR of 0.5 or more for both children and adults [36].
- Fat-Free Mass (FFM): It was calculated from the available data, which are body weight and %FM: $\mathrm{FFM}=\mathrm{total}\;\mathrm{body}\;\mathrm{weight}\;-\left(\mathrm{total}\;\mathrm{body}\;\mathrm{weight}\times \left(100\%\;\mathrm{body}\;\mathrm{fat}\right)\;\right)$
- SES: Was ascertained through an integrated metric accounting for median household income, parents’ educational attainments, and their occupational classification.

### 2.4. Statistical Analysis

The data obtained from the children and their parents were coded and entered using the Research Electronic Data Capture Platform version 15.4.2 (RedCap^®^) at the Center for Supercomputing and Visualization of UPM (CESVIMA-UPM). The statistical analysis was carried out using the Statistical Package for the Social Sciences version 29.0 for Windows (IBM SPSS Statistics, Armonk, NY, USA). A descriptive analysis of continuous data (e.g., body composition measurements, PA, sleep duration, and screen time) was carried out using mean, standard deviation (SD), and minimum and maximum values for each variable. The analysis of qualitative variables was performed using absolute frequencies (*n*) and relative frequencies (%) (e.g., weight categories and different items of the KIDMED questionnaire). Student’s *t*-tests were employed to compare means and determine statistically significant relationships between quantitative variables. For qualitative variables, the chi-square (χ^2^) test was utilized. Also, a two-way ANOVA (analysis of variance) was employed to examine the effects of sex and origin variables on a dependent variable (age, anthropometric data, and habits). Partial eta squared (η^2^_p_) is used to assess the main effects and interaction effects between the factors. The Mann–Whitney U test and Kruskal–Wallis H test were used to assess SES differences between groups and across weight status categories, respectively. For all statistical analyses, the significance was set at 0.05.

### 2.5. Missing Data

Missing data were <1% in different variables. %FM could not be measured in 0.52% of the sample. Also, some questionnaires were not fulfilled, affecting some variables such as sleep duration (SHSA) and screen time (SBQ) questions.

## 3. Results

**Figure 1:**
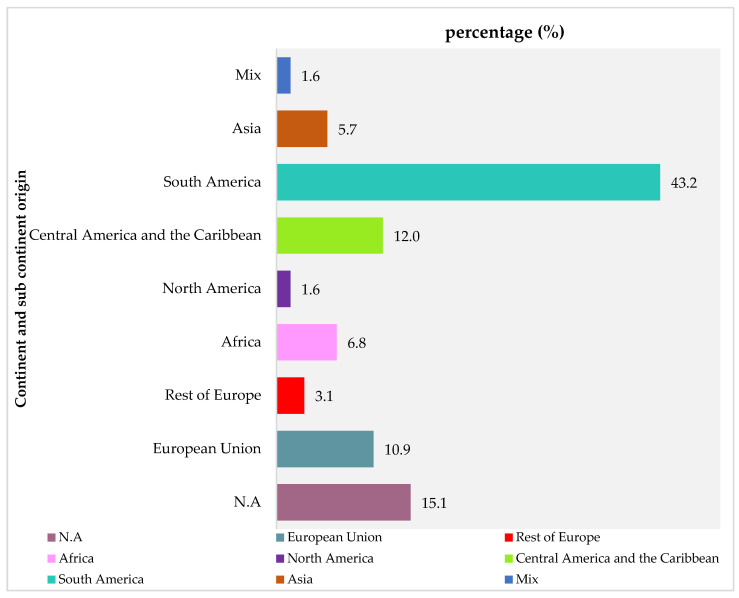
Parental origin of the migrant sample by continent and subcontinent. Note: N.A: not available.

The final sample comprised 578 academically engaged children, distributed in various primary school grades (33.4% were in third grade, 32.2% were in fourth grade, 14.4% were in fifth grade, and 20.1% were in sixth grade). Some children were excluded from the analysis (*n* = 22) due to the absence of data about their parents’ origin. Regarding the origin of their parents, 66.8% of the children (*n* = 386) had parents born in Spain, while 33.2% (*n* = 192) had parents who were immigrants. The mean age of all children enrolled in this cross-sectional study was 9.54 ± 1.19 years, with 58% of participants being male, which provided sufficient statistical power to detect meaningful effects. Figure 1 shows the different world regions of origin among the immigrant parents.

Table 1 shows the characteristics of age and anthropometric parameters of children by sex and according to the origin of their parents. Boys and girls of immigrant background had significantly higher values for height (*p* < 0.013), weight, BMI-z, FM%, WHtR, and FFM (all *p* < 0.001) compared to those of Spanish origin.

A two-way ANOVA was performed to examine the effects of child sex and parents’ origin (Spanish vs. immigrant) on six dependent variables: body weight, height, BMI-z, FM%, WHtR, and FFM. The results revealed that the interaction between the child’s sex and the parent’s origin was not significant in any case. For the body weight, a non-significant main effect of sex was observed, [F (1, 574) = 2.366, *p* = 0.125, η^2^_p_ = 0.004], and a main effect of parent’s origin, [F (1, 574) = 51.59, *p* < 0.001, η^2^_p_ = 0.082]. For the height, a non-significant main effect of sex was observed, [F (1, 574) = 1.632, *p* = 0.202, η^2^_p_ = 0.003], and a main effect of parent’s origin, [F (1, 574) = 19.83, *p* < 0.001, η^2^_p_ = 0.033]. For the BMI-z, a non-significant main effect of sex was observed, [F (1, 574) = 0.167, *p* = 0.683, η^2^_p_ = 0.00], and a main effect of parent’s origin, [F (1, 574) = 53.048, *p* < 0.001, η^2^_p_ = 0.085]. For the FM%, a main effect of sex was observed, [F (1, 571) = 34.214, *p* < 0.001, η^2^_p_ = 0.057], and a main effect of parent’s origin, [F (1, 571) = 48.956, *p* < 0.001, η^2^_p_ = 0.079]. For the WHtR, a main effect of sex was observed, [F (1, 574) = 10.584, *p* = 0.001, η^2^_p_ = 0.018], and a main effect of parent’s origin, [F (1, 574) = 33.303, *p* < 0.001, η^2^_p_ = 0.055]. For the FFM, a main effect of sex was observed, [F (1, 574) = 10.584, *p* = 0.001, η^2^_p_ = 0.018], and a main effect of parent’s origin, [F (1, 574) = 33.303, *p* < 0.001, η^2^_p_ = 0.055].

**Figure 2:**
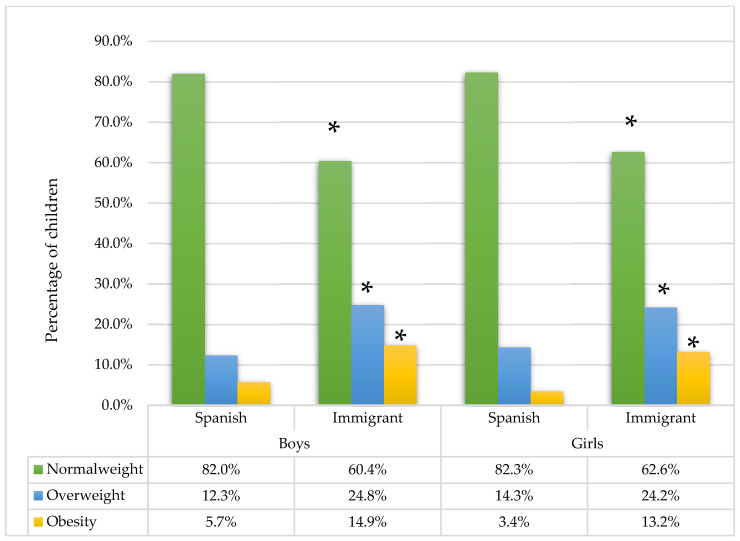
Prevalence of overweight and obesity in Spanish and immigrant children for both sexes. * indicates a significant difference between Spanish and immigrant children (*p* < 0.05).

Figure 2 shows the prevalence of OW/OB in Spanish and immigrant children. In both sexes, significantly higher prevalences of OW and OB were observed in immigrant children (*p* < 0.001).

The association between weight status (normal weight, OW, and OB) and type of school (private, semi-private, and public) was analyzed separately for native and immigrant children. Among native children (*n* = 386), no statistically significant association was found; most were classified as normal weight across all school types (private: 83.1%; semi-private: 82.7%; public: 80.2%). The prevalence of OW and OB was relatively low and similar across school types: OW was (public: 12.3%, semi-private: 12.7%, private: 15.7%), and OB (private: 1.2%, semi-private: 4.6%, public: 7.5%).

In contrast, weight status varied significantly among immigrant children (*n* = 192) by schooling type. Normal weight prevalence was highest in private schools (85.7%) and semi-private (62.7%) and lowest in public schools (53.6%). OW prevalence was highest in semi-private schools (28.4%) and public schools (24.7%) and lowest in private schools (14.3%). OB prevalence was markedly higher in public schools (21.6%) compared to semi-private (9.0%) and private schools (0%). The chi-square test revealed a significant association between the type of school and BMI category among immigrant children (χ^2^(4) = 14.42, *p* = 0.006).

Table 2 shows the time spent in front of the screen, PA, and sleep duration in the sample studied. In boys, differences were observed for time spent performing PA and in front of the screen during WD. Immigrant boys did less PA (*p* < 0.001) and spent more time in front of the screen (*p* = 0.036) than their Spanish counterparts. In girls, differences were only observed for time spent in front of the screen during WD, being higher in those of immigrant origin (*p* = 0.013).

Table 3 presents the results of the KIDMED questionnaire and a detailed analysis of the 16 items for immigrant and Spanish children. Among boys, Spanish ones consumed more baked goods or pastries for breakfast, fish 2–3 times/week, and used olive oil more frequently (all *p* < 0.05), whereas immigrant boys consumed more pasta and rice almost daily (5 times/week) (*p* < 0.001). Regarding girls, immigrants were more prone to skip breakfast and consumed pastries and fish less often (*p* < 0.05). However, they ate rice significantly more frequently than Spanish girls (*p* < 0.001). Also, a trend toward statistical significance was observed in pulses consumption (*p* = 0.055). Analyses of interactions based on sex and origin revealed significant differences in the consumption of dairy products for breakfast and fast foods and the aforementioned food items. Regarding AMD, a significant difference was observed among girls (*p* = 0.010), with immigrant girls demonstrating lower AMD compared to their Spanish counterparts. However, no significant differences were observed in the KIDMED index for either sex.

Other statistical analyses were conducted to examine the influence of lifestyle factors (PA, sleep duration, time spent in front of the screen, and KIDMED index) on BMI-z, as well as the potential effects of sex and origin, to see if a relationship exists. However, the results did not reveal any significant effect.

A series of analyses revealed significant SES disparities between native and immigrant children. The Mann–Whitney U test showed that native children had significantly higher SES compared to their immigrant peers (U = 23,221, Z = −5.55, *p* < 0.001), with higher mean SES ranks among natives (M = 300.41) than immigrants (M = 220.69).

Regarding weight status, no significant association was found between SES and weight status among native children (Kruskal–Wallis H = 1.720, *p* = 0.423; median test *p* = 0.252). In contrast, among immigrant children, SES varied significantly by weight status (Kruskal–Wallis H = 15.044, *p* < 0.001; median test *p* = 0.029), with lower SES associated with higher rates of OW/OB.

## 4. Discussion

The main findings of our study showed a significantly higher rate of OW/OB, less PA, and higher sedentarism among children of immigrant parents.

Further exploring these findings, recent data from the Spanish National Statistics Institute (Instituto Nacional de Estadística) indicate that most of the immigrant population in Spain originates from Colombia, Venezuela, and Morocco. Madrid has been identified as one of the cities experiencing significant population growth, including immigrants [37]. In this context, it is essential to consider how this demographic shift influences health trends, particularly regarding childhood obesity within immigrant communities. In Spain, over the past ten years, obesity has been more commonly seen in children of early and middle childhood ages, which raises concerns about the country’s status as one of the most obese in Europe. This is still an important health problem, although from 2011 to 2019, a reduction in the prevalence of OW, according to WHO standards, among boys aged 6, 7, and 8 years was observed in Spain, decreasing by 5.4%, 5.7%, and 5.3%, respectively [38,39].

Recent studies in Spain have yielded consistent findings with our research regarding the rates of OB among immigrant children, indicating that children of immigrant origin exhibit higher rates of obesity compared to their native counterparts across both sexes [40,41]. Additionally, several studies at the European level support these findings; for instance, a Norwegian study confirmed that migrant children were at a higher risk of being OW/OB compared to native children [42,43]. A similar trend was observed among immigrants in Italy [44]. However, it is crucial to recognize that there are exceptions to these general patterns. Notably, a study conducted in Portugal illustrated this phenomenon, revealing that the prevalence of OB among children of native and immigrant parents was found to be comparable [45].

Our results reveal a complex relationship between school type and weight status, notably among immigrant children who showed significant differences. Notably, OW/OB was more prevalent in semi-private and public schools. Public schools, particularly those located in urban regions, frequently act as the main educational settings for immigrant communities [46]. In addition, this indicates that the school environment might significantly influence health outcomes for immigrant children. U.S. data showed that meals in most schools contain total and saturated fat levels exceeding the National School Lunch Program (NSLP) and National School Breakfast Program (NSBP) guidelines [47]. Moreover, in public schools, variations in class schedules and allocated lunchtime may partly elucidate differences in obesity rates [48].

In the present study, we identified a significant percentage of immigrants from Latin American countries [49]. According to the United Nations Children’s Fund (UNICEF), the OB rate affects 30.6% of children and adolescents (approximately 49 million) in Latin America, where chronic undernutrition and OW represent a “double burden,” posing challenges for prevention initiatives [50,51,52]. The recent report from the NCD Risk Factor Collaboration group supports these data, as the highest age-standardized prevalence of OB is found in Latin America, the Caribbean, the Middle East, and North Africa [53], where most of the analyzed immigrants in the present study came from. Taking into account the prevalence data for OW/OB and the health aspects that affect children in their or their parents’ country of origin could help to better understand the determinants in this population group.

Concerning the PA, our results indicate that native children of both genders were more active than their immigrant counterparts. A systematic review by Lacoste et al. corroborates these findings, highlighting that physical inactivity is a widespread issue among children, with immigrant populations being particularly vulnerable [54]. In line with our results, a qualitative study conducted in the southwest area of Madrid found that immigrant children generally exhibited lower levels of PA [55]. Another research, including a study in the United States, also indicated that children from immigrant families were at higher risk of having lower levels of PA compared to those born in the host country [55,56]. Contrarily, one Spanish non-representative study performed mainly in Southern Spain found that immigrants were more active compared to natives. It was proposed that the sociocultural context of their surroundings could play a key role in facilitating the adaptation process for immigrant populations [57]. Multiple studies have demonstrated variations in PA patterns among different ethnic and racial groups, where in U.S. study data showed that non-Hispanic White males engage in less vigorous activity than Mexican American and non-Hispanic Black peers. Another revised literature study also found that South Asian and Black children are generally less active than White Europeans [58,59,60].

Regarding sedentary habits, in our sample, both girls and boys from immigrant origin spent significantly more time in front of screens during WD than natives, while during WK, the results were quite similar. Some authors have linked differences in non-academic screen use during WD and on WK to sociodemographic factors [61]. Other studies have shown that screen viewing and social media use were not significantly associated with immigration status; moreover, it has also been suggested that acculturation and shared environment play a significant role in shaping sedentary behavior. As immigrant children become more acculturated, their screen time and sedentary habits become more similar to those of native children [62,63].

WHO recommendations for children and adolescents are to limit screen time to two hours a day [64]. The mean values of the children included in this study are all over two hours, except for native girls during WD. Children’s screen use was already an important public health problem that has been intensified by the global COVID-19 crisis, as shown in some studies from northern Spain, where screen usage increased during confinement [65,66]. In the longitudinal analysis of the PESCA study, children from the region of Madrid increased non-academic screen time significantly from 2018/2019 to 2020/2021, being the highest between 2019/2020 and 2020/2021 in males and those aged 6 to 9.99 y [67]. It is also important to highlight that the Spanish Pediatric Association has updated screen time use in December 2024, limiting to one hour per day for children aged 6 to 12 y [68]. A study conducted by the Crecer Jugando Foundation revealed that approximately 69% of children in Spain surpass the expert-recommended maximum hours for screen exposure [69]. This was associated with health problems. The Spanish National Health Survey demonstrated that children who engage in 180 min or more of leisure screen time daily, as opposed to those who spend less than one hour, are at a higher risk of developing emotional and behavioral issues [70].

Our findings revealed patterns indicating no statistically significant differences in sleep duration between children with and without immigrant backgrounds. The average sleep duration for both groups was approximately 9 h per night during the WD and 10 h on WK. While this represents only a minimal discrepancy in minutes, previous research has indicated that immigrant children are less likely to achieve 11 h of sleep per night compared to their native peers [71]. The inquiries focused on sleep schedules (bedtime and wake-up time). The American Academy of Sleep Medicine (AASM) recommends that children between the ages of 6 and 12 consistently receive 9 to 12 h of sleep each day to maintain optimal health [72]. Alternatively, the Asia Pacific Consensus suggests a somewhat reduced sleep duration, advocating for 9 to 11 h of sleep within a 24 h timeframe [73]. In our research, as there is no consensus, we opted to adhere to these guidelines and considered a sleep duration of 9 to 11 h for children within this age group. This method seeks to honor the maximum boundaries set by the Asia Pacific Consensus while considering the diverse requirements of children.

Although our results did not reveal significant differences, other studies have reported disparities. For instance, Anujuo et al. found that 5-year-old non-native children had less sleep time compared to their native counterparts [74]. Overall, changes in children’s bedtime and waking hours were observed during and after the COVID-19 pandemic, with Kaditis et al. reporting a notable increase in sleep duration among children in North and South America compared to the preceding period [75]. However, the investigation also indicated no significant variation in sleep duration between WD and WK among children in Europe, the Middle East, North America, and South America [76]. Additionally, qualitative interviews have suggested that COVID-19 has impacted children’s sleep, with children from different racial and ethnic communities sleeping less on average [65]. Zarate et al., in the PESCA longitudinal study, also observed a significant decrease in sleep from 2019/2020 to 2020/2021 [67].

Our analysis regarding the AMD and KIDMED index revealed no difference based on children’s origin, except that immigrant girls showed a better KIDMED score than native girls. This finding contrasts with an Italian study, which reported no statistically significant differences in the KIDMED index between native and immigrant children [77]. However, it is partly consistent with the IDEFICS study, which found that children with at least one migrant parent had higher AMD [78]. When analyzing the items of the KIDMED separately, no differences were observed regarding fruit and vegetable consumption, in contrast to the study by Ruiz-Muelle et al., who found higher consumption of fruits and vegetables in children of foreign parents among schoolchildren in Almeria (Spain) [79]. Immigrant children tend to consume fewer dairy products at breakfast, consistent with previous findings among immigrant populations [80]. Moreover, discrepancies in dietary patterns among groups were observed, notably in breakfast pastry and fast-food intakes, which can vary greatly by country, setting, and culture. Consequently, migration may result in dietary behaviors that diverge considerably from those of the local population [40].

The family environment plays a crucial role in these habits. Parents commonly encourage their children to eat fruits and vegetables, and this encouragement is influenced by ethnic backgrounds, which can affect dietary preferences and habits [81,82]. Native children are indicated to consume significantly more fish and less pasta or rice than immigrants. This is in accordance with a study conducted in northern Spain [83]. These differences provide insight into the nutritional quality of the diet in both populations, particularly regarding the intake of omega-3 fatty acids, specifically docosahexaenoic acid (DHA), which is essential for cognitive development in children. This has also been observed in previous studies, showing an association between DHA intake and reduction in childhood obesity [84,85], as well as the consumption of complex carbohydrates. Immigrants may face challenges in adapting to the dietary practices of the host country [86,87]. Furthermore, the use of olive oil among the Spanish population is more prevalent in their dietary practices, which is linked to the Mediterranean diet [88,89].

The absence of statistically significant associations between lifestyle factors and BMI-z scores in our study warrants careful interpretation. While these variables are frequently cited as key determinants of childhood OW/OB [90,91], our findings align with a growing body of literature suggesting that such associations are not universally observed and may be highly context-dependent. For instance, Chen et al. (2024) found no significant association between screen time and BMI in girls when using compositional data analysis [92], highlighting the importance of both sex-specific effects and methodological approaches in shaping observed outcomes. These findings suggest that lifestyle behaviors may interact in complex ways or be moderated by unmeasured factors such as genetics, SES, or family environment [93]. Moreover, measurement limitations must be acknowledged. Self-reported data on lifestyle behaviors are subject to recall and social desirability biases, which may reduce the precision of exposure assessment.

The differences are probably rooted in SES inequalities, as it was shown that native children have notably higher parental SES compared to immigrant children [94].

Our finding aligns with broader research indicating that lower SES is associated with higher rates of childhood obesity [95], particularly for immigrant children [96]. A lower SES is associated with an increased prevalence of OW/OB. A U.S. study found that Black and Hispanic children from lower SES families demonstrate significantly greater likelihoods of these conditions [96,97].

Some limitations in our analysis that may affect these results should be mentioned, as self-reported data can be biased, and cross-sectional data do not establish causality. On the other hand, a strength of our study is that the distribution of immigrant origins in our sample closely resembles that of the region of Madrid, according to official statistical data [49]. This close resemblance increases the validity of the study, allowing for more accurate generalizations about the immigrant children population in Madrid. An additional strength is the rigorous sampling and fieldwork protocol.

## 5. Conclusions

This study reveals significant disparities in the prevalence of OW/OB, PA levels, screen time, AMD, and eating habits between immigrant and native children in Madrid. Immigrant children exhibit higher rates of OW/OB, lower levels of PA, and increased screen time during weekdays, indicating an apparently more sedentary lifestyle compared to their native counterparts.

Dietary variations significantly impact these differences, as immigrant children consume less fish and more pasta, use olive oil infrequently, go more frequently to fast-food restaurants, and have distinct breakfast habits, notably regarding dairy products and baked goods or pastries compared to their native counterparts. Our analysis underscores the need to address these differences through targeted interventions that are culturally appropriate and tailored to the specific needs of each population. Given the significant disparities in PA level and dietary habits and their potential long-term health consequences, intervention strategies should be informed by a separate analysis of the factors affecting each group. Scientific evidence supports the urgency of action to reduce these health disparities. Future research should focus on identifying the key drivers of these differences, including the cultural and socioeconomic factors that influence eating choices, to develop more effective and targeted interventions for both immigrant and native children.

## Figures and Tables

**Table 1:** Characteristics of age and anthropometric parameters of the analyzed sample.

	Total *n* = 578 (100%)	Boys *n* = 312 (54%)			Girls *n* = 266 (46%)			
		Spanish *n* = 211 (54.7%)	Immigrant *n* = 101 (52.6%)	Sta	Spanish *n* = 175 (45.3%)	Immigrant *n* = 91 (47.4%)	Sta	Sta
	Mean ± SD [Min–Max]	Mean ± SD [Min–Max]	Mean ± SD [Min–Max]	Sig ^(a)^	Mean ± SD [Min–Max]	Mean ± SD [Min–Max]	Sig ^(b)^	Sig ^(c)^
Age (years)	9.54 ± 1.19 [7.8–12.8]	9.55 ± 1.21 [7.8–12.8]	9.83 ± 1.26 [7.9–12.8]	0.063	9.31 ± 1.07 [7.8–12.3]	9.6 ± 1.2 [7.9–12.8]	0.060	0.998
Weight (kg)	34.60 ± 9.92 [20.2–91.4]	33.49 ± 9.49 [20.3–91.4]	38.90 ± 11.82 [21.9–76.4]	<0.001	31.57 ± 7.35 [20.2–55.8]	38.24 ± 10.27 [21.9–76.2]	<0.001	0.453
Height (cm)	137.55 ± 9.03 [116–173]	137.16 ± 8.44 [119.5–160]	140.01 ± 9.82 [119.6–173]	0.013	135.50 ± 8.66 [116–159.3]	139.65 ± 9.21 [121.3–162.9]	<0.001	0.407
BMI-z	0.63 ± 1.07 [−2.01–3.51]	0.37 ± 1.04 [−1.83–3.51]	0.94 ± 1.16 [−1.93–3.36]	<0.001	0.25 ± 0.96 [−1.79–2.61]	0.99 ± 0.95 [−2.01–2.92]	<0.001	0.357
FM (%)	20.83 ± 7.23 [5.7–49.7]	18.13 ± 6.28 [7.1–40.6]	21.66 ± 8.68 [5.7–49.7]	<0.001	20.97 ± 6.08 [7.2–38.7]	25.84 ± 6.66 [10.9–43.7]	<0.001	0.264
WHtR	0.45 ± 0.54 [0.34–0.73]	0.45 ± 0.05 [0.37–0.73]	0.47 ± 0.62 [0.34–0.7]	<0.001	0.43 ± 0.04 [0.36–0.61]	0.46 ± 0.06 [0.36–0.62]	<0.001	0.731
FFM (kg)	26.89 ± 5.75 [16.87–54.26]	26.94 ± 5.62 [17.76–54.26]	29.73 ± 6.81 [18.68–49.76]	<0.001	24.68 ± 4.45 [16.87–40.03]	27.82 ± 5.36 [17.56–42.9]	<0.001	0.716

BMI-z: body mass index Z-score according to IOTF; FFM: Fat-Free Mass estimated; WHtR: waist-to-height ratio; FM%: fat mass percentage; Sta: statistics analysis; Sig ^(a)^: signification inter-group (boys); Sig ^(b)^: signification inter-group (girls); Sig ^(c)^: signification of the inter-group interaction by origin and sex.

**Table 2:** Physical activity, sleep duration, and screen use in the analyzed sample.

	Total *n* = 578 (100%)	Boys *n* = 312 (54%)			Girls *n* = 266 (46%)			
		Spanish *n* = 211 (54.7%)	Immigrant *n* = 101 (52.6%)	Sta	Spanish *n* = 175 (45.3%)	Immigrant *n* = 91 (47.4%)	Sta	Sta
	Mean ± SD [Min–Max]	Mean ± SD [Min–Max]	Mean ± SD [Min–Max]	Sig ^(a)^	Mean ± SD [Min–Max]	Mean ± SD [Min–Max]	Sig ^(b)^	Sig ^(c)^
Screen time daily during the week (h/day)	1.93 ± 2.23 [0–12]	2.09 ± 2.29 [0–12]	2.76 ± 2.75 [0–12]	0.036	1.32 ± 1.38 [0–7.5]	2.02 ± 2.47 [0–12]	0.013	0.930
Screen time daily on the weekend (h/day)	3.81 ± 2.85 [0–12]	4.52 ± 2.96 [0–12]	4.51 ± 3.07 [0–12]	0.98	2.86 ± 2.17 [0–10.5]	3.26 ± 2.87 [0–12]	0.24	0.395
Physical activity (min/day)	113.85 ± 64.67 [4.29–396.43]	137.16 ± 70.75 [4.29–396.43]	106.53 ± 66.26 [12.86–300]	<0.001	99.19 ± 52.19 [15–357.86]	96.1 ± 53.97 [17.14–302.14]	0.66	0.013
Sleep duration during weekdays (h)	9.94 ± 0.99 [5.33–12.67]	9.94 ± 1.04 [5.33–12.67]	9.83 ± 1.16 [5.83–12]	0.41	10.01 ± 0.85 [6.83–12]	9.95 ± 0.93 [6.67–12]	0.63	0.748
Sleep duration during the weekend (h)	10.23 ± 1.70 [4–14.67]	9.94 ± 1.76 [4–14.5]	10.13 ± 1.83 [4–14.67]	0.4	10.46 ± 1.56 [6–14]	10.55 ± 1.55 [6–14.17]	0.63	0.766

Sta: statistics analysis; Sig ^(a)^: signification intra-group (boys); Sig ^(b)^: signification intra-group (girls); Sig ^(c)^: signification inter-group (boys and girls).

**Table 3:** Frequency of consumption of foods and adherence to Mediterranean diet in immigrant and Spanish children using the KIDMED questionnaire.

	Boys 312 (54%)					Girls 266 (46%)					
	Spanish		Immigrant			Spanish		Immigrant			
	211 (54.7%)		101 (52.6%)			175 (45.3%)		91 (47.4%)			
	Yes	No	Yes	No	Sta	Yes	No	Yes	No	Sta	
	*n* (%)	*n* (%)	*n* (%)	*n* (%)	Sig ^(a)^	*n* (%)	*n* (%)	*n* (%)	*n* (%)	Sig ^(b)^	Sig ^(c)^
Skips breakfast	15 (7.1)	196 (92.9)	11 (10.9)	90 (89.1)	0.258	9 (5.1)	166 (94.9)	11 (12.1)	80 (87.9)	0.042	0.144
Has a dairy product for breakfast (yogurt, milk, etc.)	184 (87.2)	27 (12.8)	80 (79.2)	21 (20.8)	0.067	162 (92.6)	13 (7.4)	81 (89)	10 (11)	0.327	0.013
Has cereals or grains (bread, etc.) for breakfast	143 (67.8)	68 (32.2)	68 (67.3)	33 (32.7)	0.937	116 (66.3)	59 (33.7)	65 (71.4)	26 (28.6)	0.393	0.863
Has commercially baked goods or pastries for breakfast	85 (40.3)	126 (59.7)	29 (28.7)	72 (71.3)	0.047	77 (44)	98 (56)	26 (28.6)	65 (71.4)	0.014	0.016
Takes a fruit or fruit juice every day	166 (78.7)	45 (21.3)	81 (80.2)	20 (19.8)	0.76	141 (80.6)	34 (19.4)	67 (73.6)	24 (26.4)	0.19	0.594
Has a second fruit every day	133 (63)	78 (37)	68 (67.3)	33 (32.7)	0.459	116 (66.3)	59 (33.7)	57 (62.6)	34 (37.4)	0.554	0.820
Takes two yogurts and/or some cheese (40 g) daily	162 (76.8)	49 (23.2)	70 (69.3)	31 (30.7)	0.16	143 (81.7)	32 (30.7)	67 (73.6)	24 (26.4)	0.12	0.113
Has fresh or cooked vegetables regularly once a day	134 (63.5)	77 (36.5)	75 (74.3)	26 (25.7)	0.59	121 (69.1)	54 (30.9)	68 (74.7)	23 (25.3)	0.34	0.129
Has fresh or cooked vegetables more than once a day	74 (35.1)	137 (64.9)	41 (40.6)	60 (59.4)	0.344	74 (42.3)	101 (57.7)	36 (39.6)	55 (60.4)	0.669	0.516
Consumes fish regularly (at least 2–3/wk)	159 (75.4)	52 (24.6)	50 (49.5)	51 (50.5)	<0.001	130 (74.3)	45 (25.7)	54 (59.3)	37 (40.7)	0.012	<0.001
Consumes nuts regularly (at least 2–3/wk)	93 (44.1)	118 (55.9)	41 (40.6)	60 (59.4)	0.56	86 (49.1)	89 (50.9)	41 (45.1)	50 (54.9)	0.53	0.558
Likes pulses and eats them > 1/wk	159 (75.4)	52 (24.6)	73 (72.3)	28 (27.7)	0.56	126 (72)	49 (28)	55 (60.4)	36 (39.6)	0.055	0.070
Takes sweets and candy several times every day	42 (19.9)	169 (80.1)	23 (22.8)	78 (77.2)	0.56	35 (20)	140 (80)	22 (24.2)	69 (75.8)	0.43	0.803
Consumes pasta or rice almost every day (≥5/wk)	84 (39.8)	127 (60.2)	62 (61.4)	39 (38.6)	<0.001	58 (33.1)	117 (66.9)	59 (64.8)	32 (35.2)	<0.001	<0.001
Goes >1/wk to a fast-food restaurant (hamburger)	47 (22.3)	164 (77.7)	30 (29.7)	71 (70.3)	0.15	36 (20.6)	139 (79.4)	26 (28.6)	65 (71.4)	0.14	<0.001
Uses olive oil at home	187 (88.6)	24 (11.4)	67 (66.3)	34 (33.7)	<0.001	155 (88.6)	20 (11.4)	75 (82.4)	16 (17.6)	0.07	<0.001
Adherence to Mediterranean diet											
Poor	101 (47.87)		47 (46.53)		0.674	83 (47.43)		40 (43.96)		0.011	0.142
Average	97 (45.97)		45 (44.55)			87 (49.71)		40 (43.96)			
Good	13 (6.16)		9 (8.91)			5 (2.86)		11 (12.09)			
KIDMED index											
	Mean ± SD		Mean ± SD		sig	Mean ± SD		Mean ± SD		sig	
KIDMED index	7.07 ± 2.44		6.76 ± 2.33		0.296	7.27 ± 2.07		7.04 ± 2.72		0.453	0.849

*p* value (X^2^ test). Sta: statistics analysis; Sig ^(a)^: signification intra-group (boys); Sig ^(b)^: signification intra-group (girls); Sig ^(c)^: signification inter-group (boys and girls). The KIDMED score: ≤3, poor-quality diet; 4–7, average-quality diet; ≥8, good adherence to Mediterranean diet.

## Data Availability

The ASOMAD study is now complete, and data collection has ceased. Registration of participating schools commenced in 2020, with three waves of data collection. The datasets have now been finalized and closed for analysis. Data can be requested by sending an email to the IP at marcela.gonzalez.gross@upm.es.

## Abbreviations

The following abbreviations are used in this manuscript:

AASM	American Academy of Sleep Medicine
ALADINO	ALimentación, Actividad física, Desarrollo Infantil y Obesidad
AMD	Adherence to the Mediterranean Diet
ANOVA	Analysis of variance
ASOMAD	Actividad física Sedentarismo de los niños de MADrid
BIA	Bioimpedance analysis
BMI	Body mass index
BW%	Body water percentage
COVID-19	Coronavirus Disease 2019
DHA	Docosahexaenoic acid
EU	European Union
FFM	Fat-Free Mass
FM%	Fat mass percentage
IDEFICS	Identification and prevention of Dietary- and lifestyle-induced health Effects in Children and infants
IOTF	International Obesity Task Force
ISAK	International Society for the Advancement of Kinanthropometry
KIDMED	Mediterranean Diet Quality Index for children and adolescents
NAFLD	Non-alcoholic fatty liver disease
NCD	Non-Communicable Disease
OW/OB	Overweight and obesity
PA	Physical activity
PAU-7S	Physical Activity Unit—7-Item Screener
SBQ	Sedentary Behaviors Questionnaire
SD	Standard deviation
SHSA	Sleep Habits Survey for Adolescents
SPSS	Statistical Package for the Social Sciences
UNICEF	United Nations Children’s Fund
UPM	University Politécnica de Madrid
WC	Waist circumference
WD	Weekdays
WHO	World Health Organization
WHtR	Waist-to-height ratio
WK	Weekend
